# Testing Novel Portland Cement Formulations with Carbon Nanotubes and Intrinsic Properties Revelation: Nanoindentation Analysis with Machine Learning on Microstructure Identification

**DOI:** 10.3390/nano10040645

**Published:** 2020-03-30

**Authors:** Georgios Konstantopoulos, Elias P. Koumoulos, Costas A. Charitidis

**Affiliations:** 1 RNANO Lab—Research Unit of Advanced, Composite, Nano Materials & Nanotechnology, School of Chemical Engineering, National Technical University of Athens, GR-15773 Zographos Athens, Greece; gkonstanto@chemeng.ntua.gr (G.K.); charitidis@chemeng.ntua.gr (C.A.C.); 2 Innovation in Research & Engineering Solutions (IRES), Boulevard Edmond Machtens 79/22, 1080 Brussels, Belgium

**Keywords:** artificial Intelligence, machine learning, carbon nanotubes, cement microstructure, materials characterisation, nanoanalysis, nanomechanics

## Abstract

Nanoindentation was utilized as a non-destructive technique to identify Portland Cement hydration phases. Artificial Intelligence (AI) and semi-supervised Machine Learning (ML) were used for knowledge gain on the effect of carbon nanotubes to nanomechanics in novel cement formulations. Data labelling is performed with unsupervised ML with k-means clustering. Supervised ML classification is used in order to predict the hydration products composition and 97.6% accuracy was achieved. Analysis included multiple nanoindentation raw data variables, and required less time to execute than conventional single component probability density analysis (PDA). Also, PDA was less informative than ML regarding information exchange and re-usability of input in design predictions. In principle, ML is the appropriate science for predictive modeling, such as cement phase identification and facilitates the acquisition of precise results. This study introduces unbiased structure-property relations with ML to monitor cement durability based on cement phases nanomechanics compared to PDA, which offers a solution based on local optima of a multidimensional space solution. Evaluation of nanomaterials inclusion in composite reinforcement using semi-supervised ML was proved feasible. This methodology is expected to contribute to design informatics due to the high prediction metrics, which holds promise for the transfer learning potential of these models for studying other novel cement formulations.

## 1. Introduction

Cement is considered as the most important hydraulic material in the modern construction field [1,2,3,4]. “Smart” sensing, self-healing, and self-sealing properties are in the spotlight and are engineered by nanomaterials addition [1,5], contributing also to materials design revolution for key industrial applications [1]. Since concrete is extensively studied [4], the design parameters are well-known in order to deliver suitable mechanical properties in the relevant application field. A plethora of new data are generated by characterization of novel cement formulations [6]; however, sufficient technology transfer to industry is scarce [1]. Except for the technology growth, synthesis of new materials and hybrid composite structures, the need to involve emerging evaluation methodologies is highlighted [1,7] to assist and accelerate developments [8,9]. Artificial Intelligence (AI) is a promising candidate to bridge the gap between Research and Development (R&D) and industry by establishing unbiased relations of microstructure to properties [4,6,7,8,9,10,11]. This is majorly appreciated in case of Safe-by-Design requirements regarding mechanical performance [8,12], and real-time characterization [9]. Being representative, k-means, Random Forrest (RF), Support Vector Machines (SVM), k-Nearest Neighbors (KNN) are common Machine Learning (ML) algorithms used in multiclass classification problems [4] for automated classification of microstructures [8,13]; however, these algorithms often require lot of data to train the predictive models [14]. Also, density functional theory (DFT) has been established for predicting the structure and behavior of organic (such as proteins) and inorganic (i.e., most common are calcium carbonates, oxalates, metal sulfides, etc.) crystals, which has enabled the development of ontology databases; the calculated properties of known systems and the predicted properties of hypothetical systems are included [10,14]. Similar efforts have been put in practice with experimental materials characterization (CHADA, Nanoindentation—documentation structure for characterisation data) [11]. 

Grid nanoindentation is a highly localized and non-destructive technique with high spatial resolution [6,7]. It is a method that is suitable for fast and precise characterization of construction materials as concrete, metal alloys, coatings, and composites reinforced with micro- and nano- materials [3,6,7,9,13,15,16], being one of the few techniques that can directly assess the mechanical properties at micro- and nano- level by a single experiment [6,17]. Nanomechanical properties, and especially reduced Elastic modulus (*E_r_*), are involved in materials design and various set-ups [18,19]. These applications are very sensible in regards to the applied loads and are closely related to human and environmental safety. This input can be obtained in a representative manner, since statistical nanoindentation is able to characterize a surface via a multitude of indentation events [15]. Also, the contact surface is in the same scale of the characterized phases as in case of heterogeneous cement interface [20]. Grid size is usually sufficiently large, for instance when testing concrete, to encompass the various cement phases [6]. Quantification of the constituent volume fractions provides insight of the composite as a whole entity [6,19]. Thus, the generated data are suitable for statistical representation of nanomechanical properties of the tested material [6]. 

Focusing on the case of cement composites, a lot of effort is put in regards to nanoindentation [7]. Nanoindentation technique is gaining widespread attention due to the ability to both identify and quantify cement phases [6,17,21]. In detail, pores *E_r_* and hardness (*H*) are derived by interfacial interaction with the low density (LD) boundary phases [22], and *E_r_* of hydrated phases is connected to degree of hydration [4,23]. Hardness is related to the yield strength of the hydrated cement phases, which are considered to behave as rigid cohesive plastic solids, granted that the size of interaction volume is smaller than indentation imprint. As part of this procedure, individual hydrated cement phases have been tested and processed via statistical analysis to determine *E_r_* and *H* of these phases [23,24], in order to provide the ability to connect mechanical properties to structure (density, crystallinity degree).

Portland Cement is used in every-day applications due to the low-cost, and workability. Calcium Aluminate Cements (CAC) facilitate protection from corrosion, temperature resistance, high strength, but are available at higher cost [25]. The main difference with ordinary Portland Cement lies in the active phase that is responsible for hardening due to the high aluminum content (up to 80 wt.%) [26]; monocalcium aluminate (up to 46%) is the active phase in CAC and yields into calcium aluminate hydrates (CAHs) formation instead of C–S–H [25]. In the present study, CEM I 52.5 N Portland Cement was used, which consists of 4.75 wt.% Al_2_O_3_, 19.47 wt.% SiO_2_, and 63.16 wt.% CaO; the absence of aluminate hydrates is expected, considering the phase diagram of CaO-Al_2_O_3_-SiO_2_ [26], thus C–S–H and CaOH (Portlandite) dominate [27]. Ettringite phase exists in the matrix phase mixed with Portlandite, at significantly lower ratios considering the sulfur role in ettringite formation and in the initial composition of CEM I 52.5 N [27,28]; it is expected to comprise up to ~5% combined with CAHs in the cement paste. The dominance of C–S–H is also reported in mixtures of Portland Cement and <20 wt.% of CAC, while alumina presence is restricted in the form of ettringite, calcium aluminomonosulfate, or C_4_AH_x_ [26]. A short summary of material parameters determined by nanoindentation and correlation to cement phases is provided in Table 1.

The main target of this work is to identify each cement phase nucleation dependence on nano-reinforcement by carbon nanotubes (CNTs) by clustering material parameters determined by nanoindentation initially and by identifying the hydrated cement phases with k-nearest neighbors, support vector machines, and classification tree algorithms further on. Nanoindentation data analysis is used to train models for prediction of hydrated cement phases by using raw data as input. This step is essential to overcome exhaustive probability distribution fitting approach, in order to reach unbiased conclusions about phase composition. Till now, this approach involved the use of error minimization procedures, due to the non-uniqueness of the solution [44] and depends on the selection of initial values [6], i.e., when fitting five phases with five Gaussians, a 15-dimensional (3 Gaussian parameters × 5 phases) is created and the solution represents one of the local optima [43]. Also, skewness of fitting is usually omitted by nullifying the third and fourth statistical moments in order to simplify analysis [38]. This is a task of predictive modeling, which was performed using statistics in the past decades due to insufficient computational power. Predictive modeling is rapidly growing, due to the availability of computational resources, and better results are obtained with implementation of ML [45]. Consequently, structure-property relations will enhance objectivity and knowledge-gain for decision making by the incorporation of ML. 

The matrix area of Portland Cement was selected for characterization, considering the fact that C–S–H is the major contributor in the final properties and durability of hardened cement [27]; measure nanomechanical properties in the interface transition zone to monitor phases nucleation. To our knowledge, this is the first time that cement mechanical properties are processed with ML to monitor the microstructure evolution and establish unbiased structure-property relations. Except for image classification (i.e., from Scanning Electron Microscopy, X-ray Tomography) and elemental analysis (i.e., from Energy Dispersive X-Ray Spectroscopy–EDX) no other data have been processed with ML algorithms to date, in order determine the cement and concrete hydrated phases quantitatively [4,17,31,46]. Specifically, the spatial deconvolution of Calcium(-Silicate)-Hydrates (C–S–H) of lower and higher density is not yet envisioned with AI, while there is no feedback for the interface effect induced by the neighboring clinker regions; image analysis of these regions is restricted by the color definition, which is the same for C–S–H and interfacial effect of clinker may be considered as Portlandite (pixels attributed to clusters) [31]. This fact hinders any straightforward connection of hydration progress and hydrated cement phases; this challenge is met by involving nanomechanics and supported further by the prediction metrics, which are exceeding relevant reported values for identification of individual phases of cement matrix using nanoindentation compared to image data analysis. The methodology implementation for phase identification in cement is expected to enhance analysis of testing ordinary Portland Cement by the transfer learning potential of the developed models. It is also expected to contribute to testing other formulations or concrete; similar principles are involved, along with data preprocessing, the use of classification algorithms by making several adaptations.

## 2. Materials and Methods

### 2.1. Nanocomposite Manufacturing

Specimens received, were formulated using Portland Cement (CEM I 52.5N, Lafarge Beton S.A., Paiania, ATH, Greece), CEN Standard sand (Normensand GmbH, Beckum, Germany), and distilled water (EN 196 standard). Carbon nanotubes were synthesized through Chemical Vapor Deposition method (CVD); synthesis and chemical modification process is described in detail in previous work [5,47]. The wet-mixing method was used for nanocomposites molding with 0.02%, 0.05%, 0.1%, 0.2%, 0.5%, and 1% CNTs by weight of cement (bwoc), and water to cement fraction was w/c equal to 0.5. The sand/cement ratio in mortar specimens was 3:1, while curing was performed in saturated atmosphere of 95% humidity, which was controlled by using saturated KNO_3_ aqueous solution. All the aspects of manufacturing, and hardening of nanocomposites are described in the relevant work of Karaxi et al. [5]. 

### 2.2. Grid Nanoindentation

**Figure 1:**
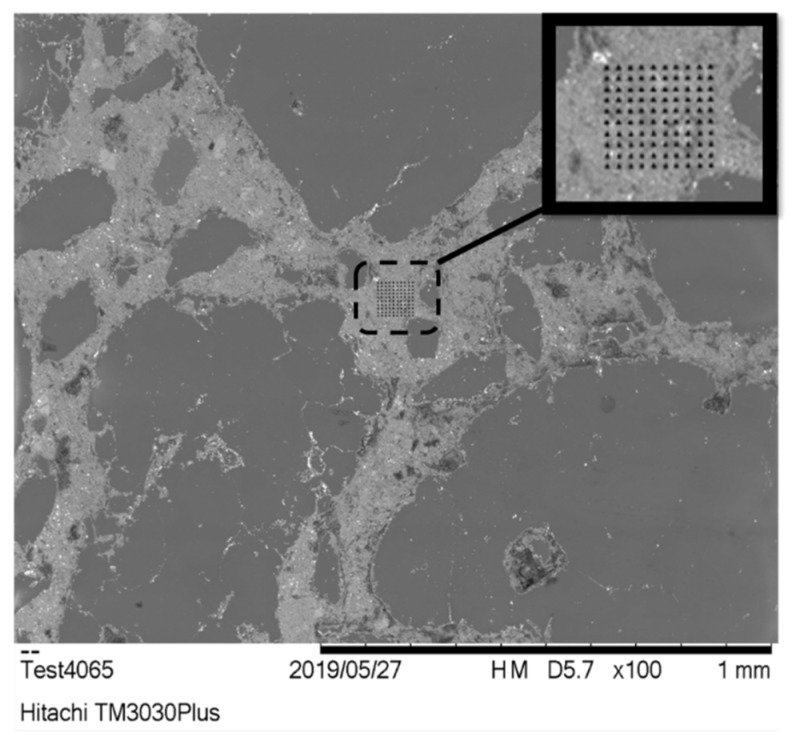
Guide for the eye: nanoindentation grid (in inset) in the interface area of Portland Cement.

The nanoindentation tests were performed using a Hysitron (Minneapolis, MN, USA) TriboLab^®^ Nanomechanical Test Instrument equipped with a Berkovich diamond indenter (average radius 100 nm), which allows the application of loads from 1 to 30,000 µN and records the displacement as a function of applied loads with a high load resolution (1 nN) and a high displacement resolution (0.04 nm). Details about the instrument and the experimental setup have been presented elsewhere [16]. Maximum indentation depth was set at 200 nm in accordance to restrictions to satisfy the separability scale condition (*d/10 << h_max_ << D/10*, *d* and *D* stand for the characteristic sizes of the largest heterogeneity) of cement phases [17,29,32,38]. Prior to indentation, the area function of the indenter tip was calibrated in a fused silica, a standard material for this purpose. All nanoindentation tests were conducted in a clean area environment with 45% humidity and 23 °C ambient temperature with displacement feedback control closed loop. All the aforementioned details comply with all nano range testing specifications as reported in ISO 14577-1:2015 for instrumented indentation. The volume of interest included the matrix region of cement (C–S–H, CH, and interface) similarly to Figure 1. 

Specimen preparation follows flat surface requirements for testing; a smooth surface was obtained by a wet polishing procedure using ethanol [48]. The used granulometry was a sequence of 400, 1000, 1200, 2000, and 4000 SiC grinding papers for 10 min each, by Struers LaboPol-2 grinding, lapping, and polishing apparatus. The specimens were dried before testing at 125 °C to prevent further progress of hydration reactions, in order to remove humidity and water captured within crystal lattice [49]. Nanoindentation testing was performed with adequate spacing of 5 μm to avoid any indentation-to-indentation interaction [7] and characterize individual cement phases [35,41]. The indenter was selected to probe at 200 nm of displacement. Material parameters determined by nanoindentation were measured via fitting with Oliver–Pharr model using the elastic response within the region of maximum load of unload curves [50]. 

### 2.3. R Language

R Language was implemented in R studio. R (version 3.6.0, R Core Team (2019). R: A language and environment for statistical computing. R Foundation for Statistical Computing, Vienna, Austria. URL https://www.R-project.org/) is an open-source software to use and provides a coherent, flexible system for data analysis. k-means algorithm was used for unsupervised data clustering. This was implemented as a labeling step to prepare data for classification. Phase correlation to material parameters determined by nanoindentation is usually a part of statistical PDA analysis, which is also performed via R for comparison. Then, the labeled data were used as a library to train classification models of RF, SVM, and KNN in order to evaluate the best predictive performance. All statistical calculations were performed using 64-bit Windows 10 Home (Intel ^®^ Core™ i5-8250U CPU @ 1.60 GHz, 1801Mhz 4 Cores, 8 Logical Processors and 8.00 GB RAM). Computational time did not exceed a few seconds in each case. The session info and the R-packages are summarized in Table A1.

### 2.4. Statistical Metrics

In order to evaluate the prediction efficiency of the trained models, statistical metrics are involved. Accuracy, Precision, Recall, F1 were exported in each case [4,51,52,53], after tuning each model to the optimum in regard to accuracy by performing grid parameterization. Accuracy accounts for overall model accuracy. These metrics are maximized when the model does not generate false positive or false negative predictions as can be observed below:

(1) $$Accuracy=\frac{TP+TN}{TP+TN+FP+FN}$$

(2) $$Recall=\frac{TP}{TP+FN}$$

(3) $$Precision=\frac{TP}{TP+FP}$$

(4) $$F1\;Score=2\times \frac{Precision\times Recall}{Precision+Recall}$$

True positives (*TP*) denote correct classifications of Portland Cement phases (positive sample), true negatives (*TN*) denote correct classifications of negative samples, false positives (*FP*) denote the incorrect classifications of negative samples into positive samples, and false negatives (*FN*) denote the positive samples incorrectly classified into negative samples. Recall is the percentage of positive samples which are correctly classified. Precision is the percentage of positive samples out of the sum of positive observations. F1 score is a metric to evaluate the model ability to classify (best value: 1). Micro-metrics are expected to obtain the same value because there is only one class associated with each instance [52]. MacroAvgPrecision, MacroAvgRecall, MacroAvgF1 are mean values for overall model metrics. MicroAvgPrecision, MicroAvgRecall, MicroAvgF1 are metrics derived by the sum of the individual true positives, false positives, and false negatives of the system for different sets.

## 3. Results: Prediction of Portland Cement Composition

### 3.1. Data Preprocessing

Nanoindentation raw data obtained from eight different Portland Cement specimens (reference and reinforced with CNTs) were merged using R language to a total of 790 indentations. Data were normalized in order to export the correlation matrix between the total nine variables. The recorded variables are:

- *h_c_*: is the contact depth,
- *P_max_*: is the peak load during a single nanoindentation event,
- *S*: is a continuous variable and represents the stiffness of a material,
- *A*: is the contact area
- *E_r_*: is the reduced elastic modulus after fitting the Oliver–Pharr model
- *H*: is the hardness after fitting the Oliver–Pharr model
- *A*, *h_f_*, *m*: are the power law coefficients

**Figure 2:**
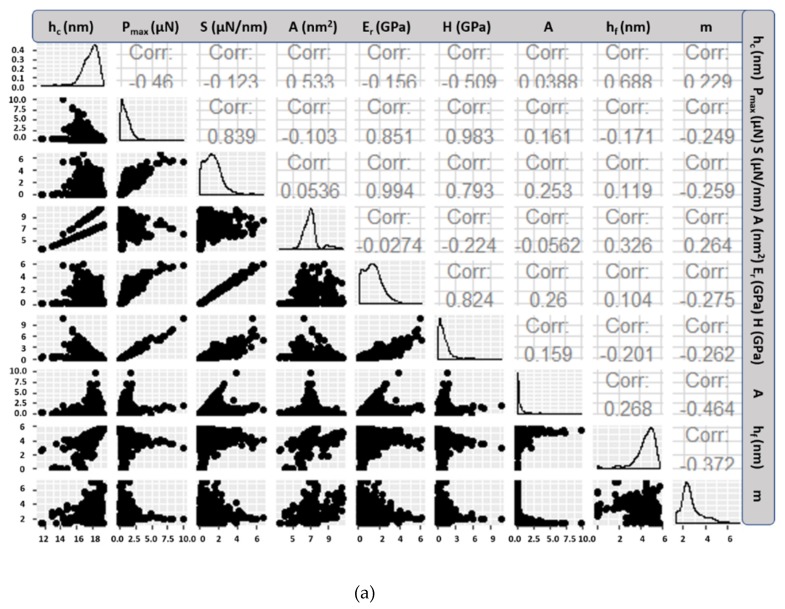

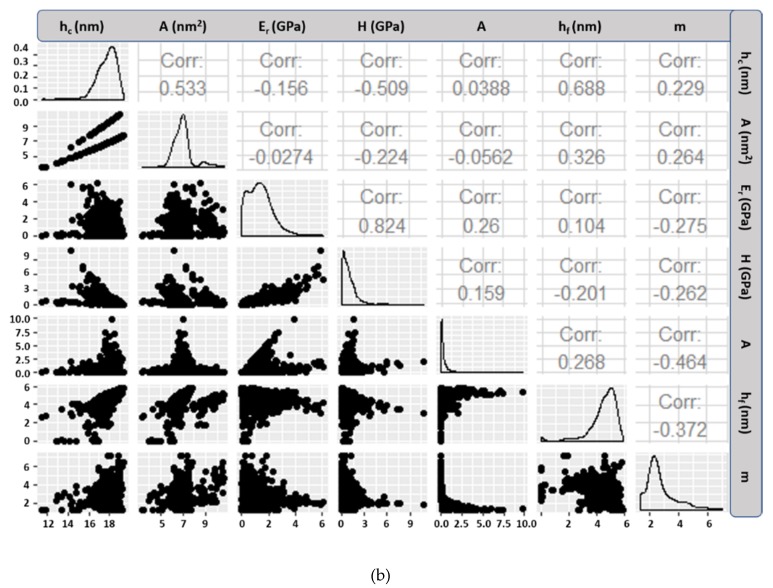
Correlation matrix of nanoindentation data prior (**a**) and after (**b**) data preprocessing. The scatter plots correspond to each combination of variables by two.

*h_c_* (nm) ranged between 11 and 20, *P_max_* (μN) ranged between 0 and 11, *S* (μN/nm) between 0 and 7, *A* (nm^2^) ranged between 3 and 11, *E_r_* (GPa) between 0 and 6.5, *H* (GPa) between 0 and 10, *h_f_* (nm) between 0 and 6, *A* between 0 and 11, and m between 1 and 7.5. The use of variables without strong correlation is necessary to avoid overfitting during training the classification models, and thus optimize computational time requirement. Thus, variables with positive or negative Pearson correlation exceeding the value of *R^2^ = ±0.90* were excluded from analysis due to very strong correlation (Figure 2) [54]. A very strong correlation (>0.90) of variables means that the model based on training data provides a very accurate fit, which also complicates the patterns behind the fitting (termed as overfitting) and this usually hinders prediction of unknown testing datasets. 

All the other variables were retained as special features of each cement phase will contribute to the identification problem during training of classification models. Then data were split to 80% train and 20% test datasets for classification models training and testing similar to [55].

### 3.2. Data Labeling

**Figure 3:**
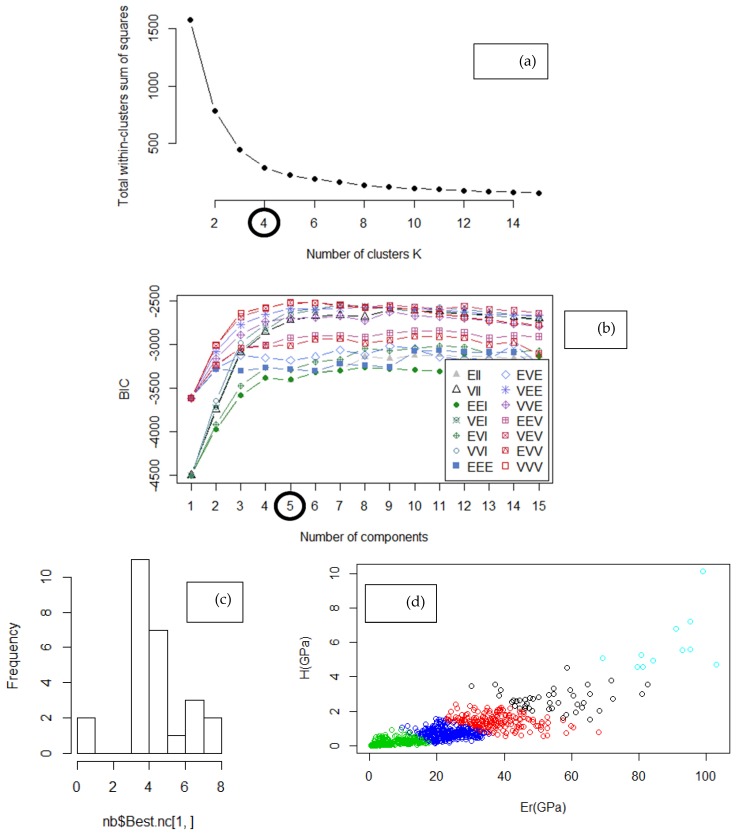

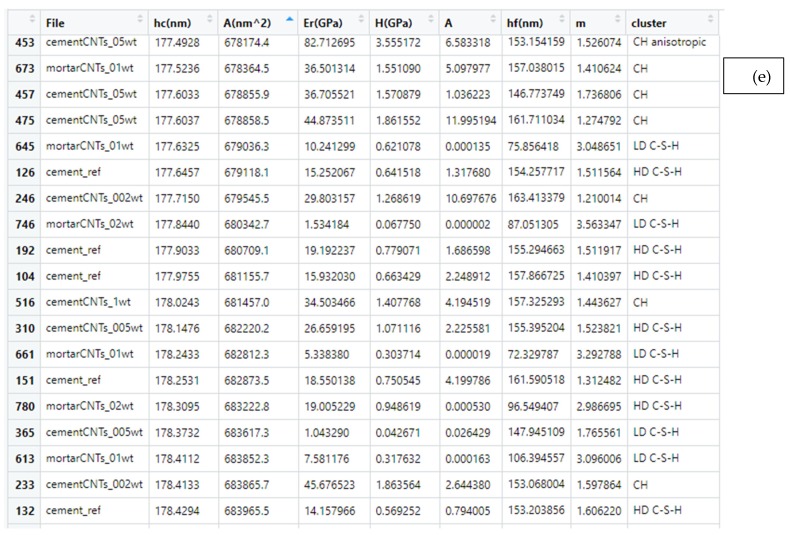
Determination of optimum number of clusters (noted in circle for **a**, **b**) with (**a**) the elbow method, (**b**) Bayesian inference criterion, (**c**) Humbert criterion, and (**d**) *H* vs. *E_r_* plot of clustered data by k-means algorithm, (**e**) labelled dataset. Symbols in (**b**): “*EII*”: spherical, equal volume, “*VII*”: spherical, unequal volume, “*EEI*”: diagonal, equal volume and shape, “*VEI*”: diagonal, varying volume, equal shape, “*EVI*”: diagonal, equal volume, varying shape, “*VVI*”: diagonal, varying volume and shape, “*EEE*”: ellipsoidal, equal volume, shape, and orientation, “*EVE*”: ellipsoidal, equal volume and orientation, “*VEE*”: ellipsoidal, equal shape and orientation, “*VVE*”: ellipsoidal, equal orientation, “*EEV*”: ellipsoidal, equal volume and equal shape, “*VEV*”: ellipsoidal, equal shape, “*EVV*”: ellipsoidal, equal volume, “*VVV*”: ellipsoidal, varying volume, shape, and orientation.

K-means algorithm was used to perform unsupervised ML and correlate material parameters (determined by nanoindentation) to Portland Cement phases. The number of clusters should be selected with pure probabilistic basis [46]. Thus, a multitude of criteria were incorporated. By applying the elbow method, the Bayesian inference criterion, and the Humbert criterion, the optimal number of clusters was estimated (Figure 3) [46,56]. Knowledge about the physical problem (Table 1) is still necessary since the aforementioned criteria demonstrate a different optimum number of clusters. The background of cement microstructure that is expected to be present within the characterized region (Figure 1) is solid. Taking into account that clustering within this study is performed for assigning labels on nanoindentation data in the framework of semi-supervised Machine Learning [57], the expected number of phases is 5 in the interface region of Portland Cement.

As depicted in Figure 3, it is proposed that optimum number of clusters is 4 (Figure 3a), but this is not confirmed by Bayesian inference criterion (BIC, Figure 3b) [46]. The maximum BIC value provides evidence for the number of existent constitutive phases in the dataset [2,6,58], which is number 5. The Humbert criterion correlates the highest frequency to the optimum number of clusters. Since 4 clusters are not sufficient to describe the number of cement phases, the next acceptable candidate is 5 clusters as indicated by the physical problem (Table 1). These clusters are attributed to LD C-S-H, including their presence in (gel) pores network (with lower stiffness), high density (HD) C-S-H, CH and its anisotropic configurations, and clinker interface (Figure 3e). At high w/c ratios (typically 50:50 similar to the present study), UHD C–S–H is absent and CH phase is formed (only), since their nucleation mechanism is antagonistic [24,42].

### 3.3. Probability Distribution Analysis-PDA

The most common approach to analyze nanoindentation data, especially in case of multiphase materials, is the application of Probability Density Function (PDF) that is intuitive regarding the identified histogram of material parameters (determined by nanoindentation) and the density plot. As a result, individual phases can be distinguished in the graph. In order to allocate the data in cement phases, a mixture model fit was selected based on Fraser–Suzuki function. The gain compared to conventional Gaussian-type normal distribution used in PDA is that the Fraser–Suzuki function allows for asymmetry [59], and that it is possible to provide a more appropriate fitting considering that neither measurement or the material are perfect [38,60]. The equation that describes the phase contribution to the overall PDF is presented below [59]:

(5) $$PDF=p_{i}\exp\left(-\frac{ln2}{s_{i}^{2}}{ln}^{2}\left(1+2s_{i}\frac{E_{r}-E_{m,\;i}}{d_{i}}\right)\right)$$

where *E_r_* is the reduced elastic modulus in GPa, p_i_ is the peak value of probability for each individual phase, *s_i_* is the skew of the fitting curve, *E_m,i_* is the mean reduced elastic modulus value of each individual phase, and *d_i_* is the width, an input value correlated to deviation from the mean value for each phase. 

**Figure 4:**
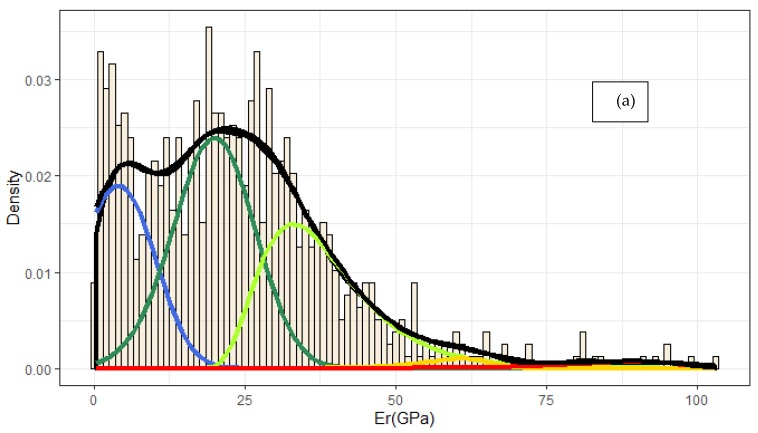

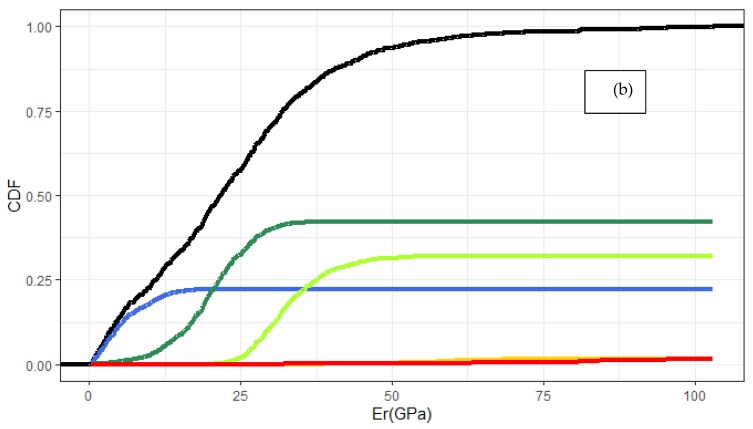
(**a**) Deconvolution of Portland Cement phases using Fraser–Suzuki function to fit nanoindentation data. The parameters are presented in Table 2. (**b**) the respective cumulative distribution fitting. Colors represent: blue: low density (LD) Calcium(-Silicate)-Hydrates (C–S–H.) green: high density (HD) C–S–H, light green: CH, orange: anisotropic CH, red: clinker interface.

The phases have been identified by the use of nanoindentation to plot histograms of material parameters as for instance *E_r_* or *H* [3,7,15,17,29,31,37,38,39,43] and by using the number of peaks in the density plot to manually select the number of phases to deconvolute (Figure 4a) [3,29,32,43]. All parameters of bin size, volume fraction, and initial mean values are all determined by the analyst prior to using PDF for fitting the data (Figure 4a) [3,38,39]. Nanoindentation data are preprocessed by the analyst to clean the error values, such as indentations that overcame the predefined maximum depth of 200 nm. The model estimated a total of 20 (4 × 5) parameters in order to describe the 5 components of Portland Cement phases. Then minimization of PDF is evaluated whether the results of mean values, standard deviation, and volume fraction are descriptive for the analysis. If not, then initial values are readjusted and analysis is performed again [2,24,31]. In order to minimize the deviation between theoretical and empirical PDF, two approaches are adopted: least squares estimation (LSE) and maximum likelihood estimation (MLE–bin size is not selected manually) [2,24,61]. Starting values were selected based on nanoindentation studies on cement phase deconvolution [41]. For this purpose, an application for quick PDF was developed within this work [62]. The optimum parameters were selected based on the theoretical PDF, analyst decisions, and are presented in Figure 4. The probability distribution was measured by the integral of Equation (5). The respective fitting of cumulative distribution function is presented in Figure 4 for completeness. It was considered purposeful to compare phase identification using PDF to k-means clustering result. k-means clustering is unbiased by the analyst choices since the physical problem is well studied, and accepts input from multiple variables (in this case *E_r_* and *H*).

By using the Shiny app, which was developed within this work [62], and by performing the PDA, it is evidenced that the probabilities of Portland Cement phases deviate among individuals’ judgements. Thereby, the authors recommend an alternative route than the PDF deconvolution procedure itself as a method for estimation of cement phases. The foundation is to create a database with labelled data, unbiased by the analyst, and use this source to classify new unlabeled data. The statistical summary is presented in Table 2. The mean values for *E_r_* are acceptable (Table 1) by both analyses; however, the variation of the mean values is minimized only in case of k-means. Also, high stiffness phases can be determined almost identically by using these two approaches. This fact may be attributed in high stiffness contrast between CH and clinker interface, that allows sufficient separation of data. The lower stiffness phases in cement matrix demonstrate a dense scattering of values, and the range of *E_r_* to describe individual phases contains data that could be attributed to a different chemical structure. Thus, it can be understood that phase identification of cement phases is not a one-dimensional problem. As a result, the correlation of chemical structure to material parameters is more reliable when using the k-means approach.

### 3.4. K-Nearest Neighbors-KNN

**Figure 5:**
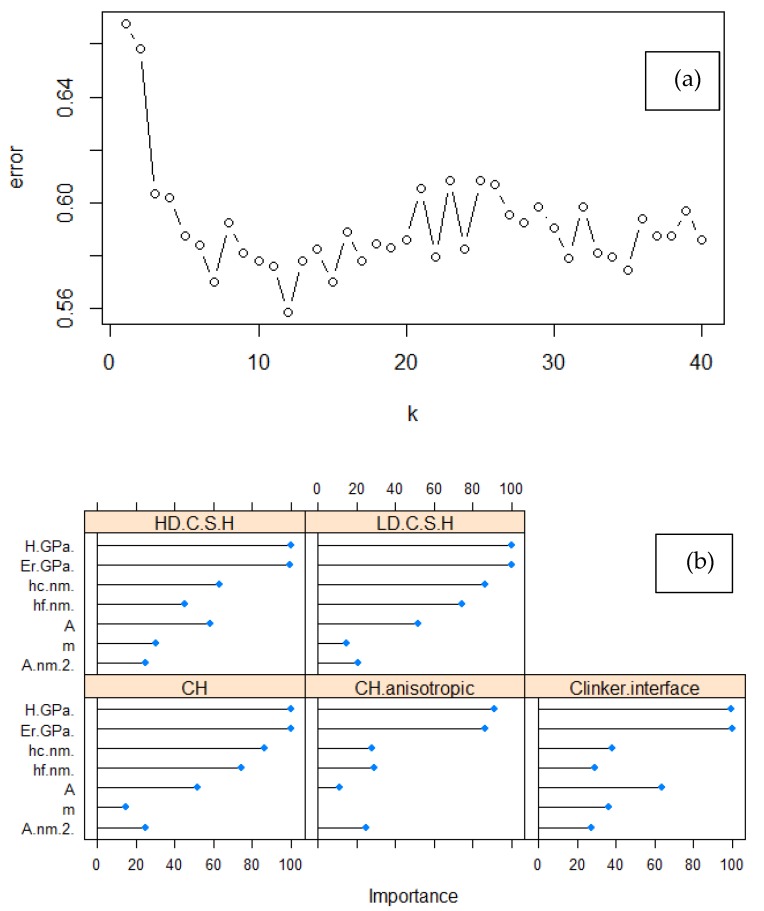
KNN model (**a**) hyperparameter tuning to identify the optimum number of k nearest neighbors, and (**b**) variables importance.

**Figure 6:**
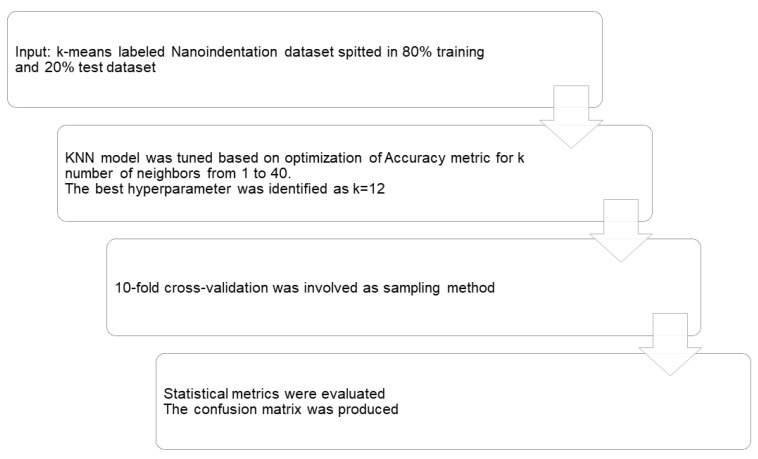
Graphical representation of the stepwise refinement process of K-Nearest Neighbors (KNN) classification for a sort program development.

K-Nearest Neighbors (KNN) algorithm was utilized since it is possible to perform multiclass classification of data. It is based on the simple principle of distance calculation between data points [14], based on Euclidean distance in the present case, and recognition of classes based on similarity. The probability of a data point to be classified in a specific group of points is solely dependent on minimization of distance between the reference data points [4]. Thus, the result of KNN classification is highly related to the selection of the integer parameter “k”, which accounts for the number of nearest neighbors [14]. Tuning was performed as presented in (Table A2, Figure 5), in order to minimize error value. Dispersion value accounts for the radius of the largest Euclidean ball containing no points. A graphical representation using KNN approach is presented in Figure 6. The variables importance that were involved from nanoindentation raw data demonstrate that predictions are mainly dependent on material parameters *E_r_* and *H*.

Since the analysis is performed in the whole data entity, the majority data representation by LD C–S–H class provides sufficient information for predictive performance, resulting in an F1 score of 0.975. However, KNN algorithm is highly affected by the imbalance in the dataset classes, as can be envisioned in Table 3. Although the prediction of HD C–S–H phase is acceptable, the F1 scores (maximum value is 1 for excellent prediction) for clinker interface and anisotropic CH phases demonstrate some drawbacks. This is also attributed to lack of data in these classes as demonstrated in the confusion matrix (Table A3), and also the imbalance in the training set deteriorates the prediction-ability. Moreover, even though micro and macro metrics overcome the random guess predictive ability of the proposed KNN model, still three classes suffer by inaccurate predictions. This is attributed to contribution from the majority of data, which are allocated in LD and HD C–S–H phases (imbalance) and increase the average of each equally weighted metric in case of micro, and in a lesser extend in macro. This is connected with total number of *TP*, *TN*, *FP*, *FN*. Large classes significantly affect the micro-metric values, and thus these values are closer to the metrics of LD and HD C–S–H phases.

### 3.5. Random Forrest-RF

**Figure 7:**
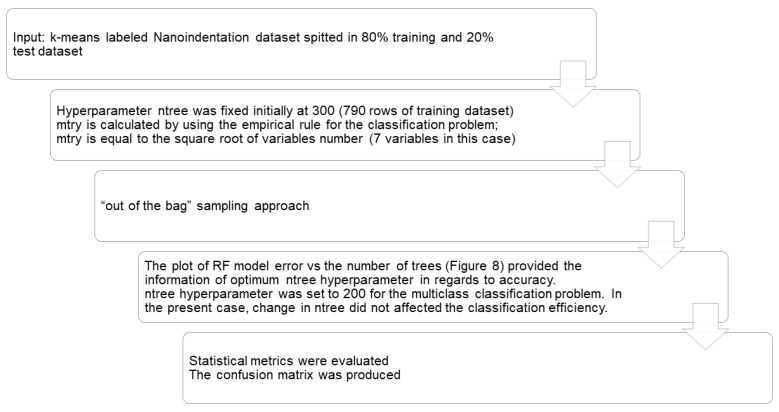
Graphical representation of the stepwise refinement process of Random Forest (RF) classification for a sort program development.

Random Forest (RF) is a decision tree-based algorithm, which can perform both regression and classification [4]. It is a multifaceted algorithm that is usually used when there is uncertainty whether algorithm may be a more appropriate fit for the classification problem [4]. By the application of the algorithm, a tree is structured to correlate consecutive choices or outcomes, with branches indicating that each option is mutually exclusive [14]. RFs are sensitive to the training dataset in their predictions; thus, sampling method is important in reproducibility of results. Bagging method was adopted in present case, which maintained the same bin size of data points within each tree formation by replacement, and consequently the results are not dependent on the gradual reduction in the sampling size. Finally, data are classified based on the majority of votes by the classification tree [4]. The graphical representation of RF is summarized in Figure 7. 

**Figure 8:**
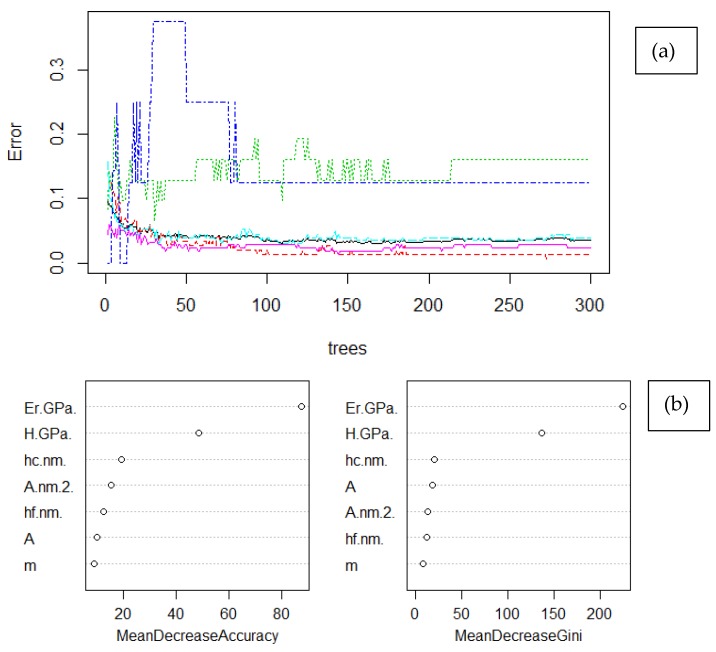
Random Forest (RF) model: (**a**) error plot vs. number of trees. Colors in figure account for; red is the LD C–S–H phase, magenta is the HD C–S–H phase, with light blue is the CH phase, with green is the anisotropic CH phase, and blue is the clinker interface, and (**b**) variables importance.

RF model demonstrated higher adaptivity on the training dataset and provided sufficient classification metrics. Phase identification is mainly dependent on *E_r_* and secondarily on *H*, while the other variables minorly affect the prediction accuracy as depicted in Figure 8. RF model lead to F1 scores that exceeded 0.909 in all occasions (Table 4), namely LD and HD C–S–H phase, CH phase and its anisotropic configuration, and the interface to clinker. The overall model accuracy exceeded 97.5%, and holds promise for successful identification of unlabeled data. The weakness of the RF model is identified in Precision metric—in order to improve precision another algorithm should be used, in order to generate less false negatives during prediction in test dataset. High recall is the reason why the MacroAvgRecall is higher than MicroAvgRecall. The rest of macro- and micro-metrics follow the expected trend; therefore, macro-metrics are closer to minority classes and micro-metrics are closer to majority classes. No bias was observed in the confusion matrix (Table A4) in the prediction of majority class.

### 3.6. Support Vector Machine-SVM

**Figure 9:**
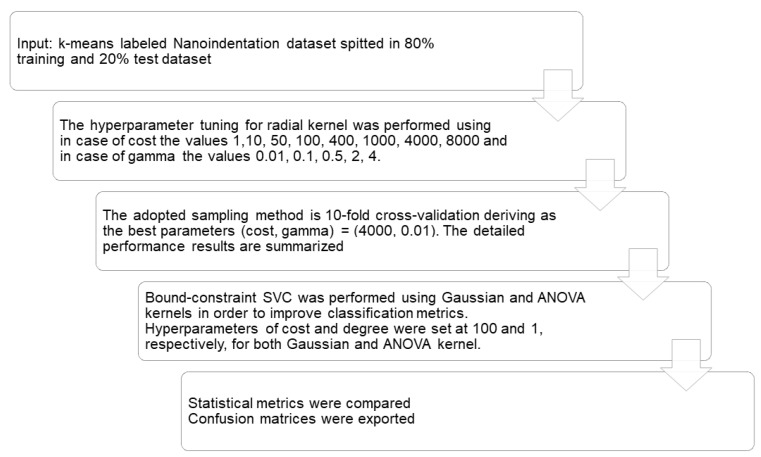
Graphical representation of the stepwise refinement process of Support Vector Machine (SVM) classification for a sort program development.

Support Vector Machine (SVM) is based on statistical learning theory and consists of heuristic algorithms [4]. Multi-dimensional data are fitted using a kernel function to provide an analogous representation, which simplifies the classification process. The kernel suitability is determined by the similarity of the kernel function to the representation of data in a high-dimensional space [14]. The hyperplane function is the boundary that separates the data for classification [8]. The implementation of SVM is summarized in Figure 9.

Support Vector Machine classification was first applied using a simple radial kernel function (Table 5). Hyperparameter tuning (Table A5) was performed to find optimum values for kernel complexity by cost hyperparameter adjustment. Similarly, optimum gamma value was selected, which is correlated to the retention of “bad” data points during training of the model (the higher the gamma value, the “bad” values are disregarded). However, even after tuning, the precision in anisotropic CH still suffered, and thus a lowered F1 was observed comparatively to other phases. Consequently, another kernel function should be considered to separate effectively the data into correct classes. 

To handle prediction weakness of Anisotropic CH class, Gaussian kernel function was considered (Table 6). After several trials, the optimum values of cost and gamma were identified as 100 and 1, respectively. The kernel function change was effective, as Recall metric was higher compared to radial kernel, but no effect was evidenced in Precision. Thus, F1 score demonstrated an increment. The higher prediction efficiency of Anisotropic CH class was accompanied with reduction by 1 and 2% in the F1 score of LD and HD C–S–H phase, respectively; still, F1 score is considerably high. Macro- and micro-averaged metrics demonstrated the same trend as in case of radial kernel SVC.

In order to find out if another kernel function could overcome the prediction efficiency of Gaussian kernel, and also RF performance, ANOVA kernel function was considered (Table 7). Separability of data further improved the F1 score, which overcame 95% in all categories, and F1 score was improved by 1% for CH class, and by 4.5% for anisotropic CH. This improvement in case of Anisotropic CH class was influenced by Precision increment by using ANOVA kernel. Thus, even though the overall model accuracy is the same for all RF, radial SVC kernel and ANOVA SVC kernel, ANOVA approach could be more sensitive in the correct prediction of an unlabeled nanoindentation event (Table A6, Table A7, Table A8). Consequently, it is considered as a model that is unbiased by the data imbalance and favors dealing effectively with Portland Cement phases classification problem by achieving higher individual precision compared to the aforementioned models. This conclusion is further supported by the higher observed values of micro-metrics, with MicroAvgPrecision exceeding MacroAvgPrecision, and by achieving a very similar F1 value for both metrics.

## 4. Discussion

Till now, the most common approach when studying the cement hydrated phases with nanoindentation included Probability Distribution Analysis. This approach was based solely on deconvolution by Gaussian fittings, which suffers from the non-uniqueness of the solution. The number of peaks in the density plot, and thus the number of Gaussians, create a multidimensional space when measuring the parameters of the solution, and this approach suffers by the existence of more than one global optima. As a consequence, the analysis of the same nanoindentation data may vary amongst individuals. Moreover, third and fourth statistical moments are nullified by assuming zero skewness and using Gaussians to fit data, which introduces another factor for error evolution. Within this study, a practical approach in PDA is summarized by introducing skewness by the incorporation of Fraser–Suzuki equation for fitting. The input parameters of *p_i_*, *s_i_*, *E_m,I_*, *d_i_* are useful for later analysis with integrals and calculation of the composition percentage of each individual phase. 

Machine Learning came up as a more efficient route to deal with the multivariate problem of nanoindentation raw data. Implementation of an unsupervised ML algorithm for unbiased determination of cement phases was demonstrated using k-means clustering. Unsupervised phase identification showed the magnitude of variation in microstructures volume fraction. This is an introductory step for labelling the test data. Labelling is useful to perform quick evaluation of cement composition by training supervised ML models for performing quality control of the synthesized or nano-enforced cementitious structures. This approach known as semi-supervised Machine Learning is also unbiased, can be reproduced, and combines multidimensional features. Inclusion of seven variables enables establishing structure (cement phase)—property (parameters: *E_r_*, *H*) relations to contribute in reinforcement identification due to possible enhanced propagation of hydration and nucleation aided by nanomaterials. This reinforcement is envisioned especially in cement interface (or matrix) and the compositional changes in LD, HD C–S–H, and CH phases, considering that better interfacial properties will improve overall performance. 

**Figure 10:**
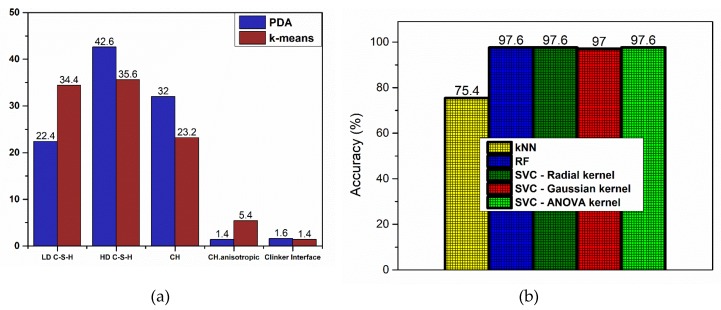
Summary of major results: (**a**) volume fraction (%) as measured by PDA vs. k-means, (**b**) represent the comparative histograms of Precision, Recall, and F1-score, respectively.

Cement phase identification, which was implemented using two methodologies, a PDF fitting with skewness and unsupervised ML algorithm of k-means enabled to identify the strengths of each approach directly. In the first case, PDF was applied based on Fraser–Suzuki function in order to improve fitting results as asymmetry is enabled. This implementation is also available in the shape of Shiny app to fit *E_r_* nanoindentation data using R language [62]. Two significant aspects should be pointed out. Firstly, the presented case required 5 PDF fittings, which means that a 20-dimensional space (5 PDFs × 4 parameters) is created for the problem solution. Thus, the proposed fitting falls within one of the local optima set of solutions. As a result, it can be understood that another solution may be chosen by another individual. Secondly, the solution in PDA is about the single-parameter problem (here: reduced elastic modulus). The deviation is high, as expected, compared to k-means clustering approach, which incorporated more variables to provide the microstructure clusters. As demonstrated in Figure 10a, the PDA predictions were biased to HD C–S–H and CH phases. This deviation was not encountered for the clinker interface, as both methodologies lead to similar results in case of *E_r_* value, possibly due to the low population of available data. In this direction, k-means correlated material parameters to cement chemical structure using both parameters *H* and *E_r_* as input. Consequently, k-means was used for unbiased labelling of the nanoindentation data in order to feed the data for multiclass classification and perform semi-supervised Machine Learning. In conclusion, identification of cement phases using nanoindentation data is improved when it is approached as a multivariate problem with Machine Learning, which was expected in principle [45].

**Figure 11:**
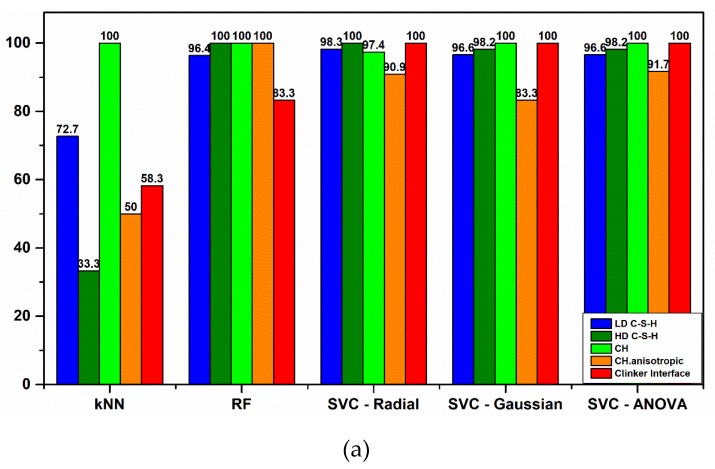

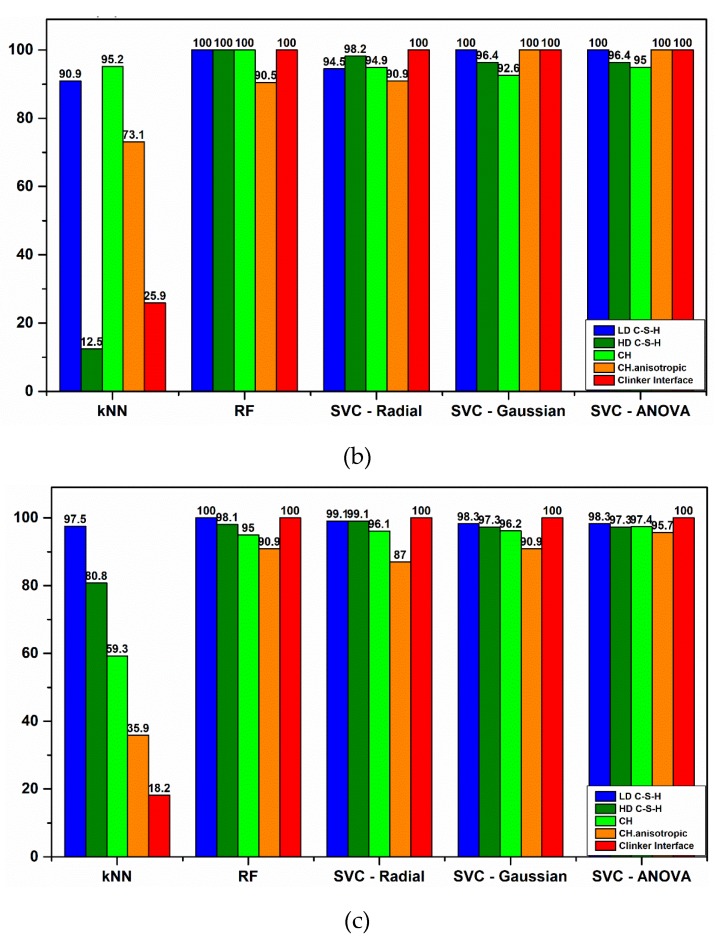
Summary of major results: (**a**–**c**) represent the comparative histograms of Precision, Recall, and F1-score, respectively.

In another case, k-means clustering has been used in order to predict the nanoindentation response of a given location in FCC single crystals [51], with overall accuracy to reach 90%. In this work, multiclass classification was performed using common algorithms of KNN, RF, and SVC reaching a maximum accuracy of 97.6% (Figure 10b). In detail, KNN model was trained in order to predict the classes in the test dataset. However, the model performance was not adequate even after tuning of hyperparameters, especially for the high-stiffness phases identification, reaching a minimum F1 score of 0.18 (Figure 10e). This result could be possibly correlated to the low population of data for these cement phases and imbalance in the population amongst cement microstructure classes [53]. On the other hand, all other algorithms (RF and Radial, Gaussian, and ANOVA SVC kernel types) after being properly tuned were able to use all seven variables to correctly classify nanoindentation events to Portland Cement phases. A minimum score of F1 = 0.87 in case of Radial SVC kernel was achieved and the highest minimum-class predictive score of F1 = 0.96 was accomplished with ANOVA kernel. Also, with ANOVA kernel the weakness of SVC in Precision metric was overcome. Although RF demonstrated the same number of misclassifications in the test dataset as ANOVA SVC kernel, in fact, misclassifications are not accumulated in a single category when using ANOVA, which is preferable compared to Random Forrest. The key in finding the best possible algorithm for each scenario is identified in testing a variety of classification algorithms in order to find the right balance between Precision and Recall metrics, since often it is challenging to keep both high in value (Figure 11a–c). Even if Random Forrest provided the highest Recall in HD C–S–H and CH phases, it was preferred to sacrifice Recall in favor of achieving higher Precision in classification of those two phases, in order to reduce misclassification error of the trained models in future input of unseen data and achieve high prediction metrics in all hydrated cement phases. 

## 5. Conclusions

This study aimed in the implementation of an enhanced practice for analysis of cement microstructure with nanoindentation testing. Step-by-step methodology of data preprocessing, labelling, and classification is summarized in order to enhance interlaboratory reproducibility of the results. It is important to note that nanoindentation protocol for mapping majorly affects the usability of data for phase identification and highlights the necessity for establishing good practices in testing cement formulations. A common approach in characterization protocols is essential to enable data exchange and further developments in characterization methods. Semi-supervised Machine Learning was implemented due to the enhanced efficiency in predictive modeling of microstructure. In principle, Machine Learning exceeds traditional statistics for predictive modeling. This is derived by the inclusion of more variables, and thus data, to pattern relationships in the labeled data. The fitted patterns become more complex and contain more information for the microstructure classes. This is a gain compared to traditional statistics for prediction of cement phases, which in case of Probability Distribution Analysis, uses a single parameter for identification. Increased complexity in relations of nanoindentation data and cement microstructures enhances the level of prediction accuracy when testing other formulations not previously used for training Machine Learning models. High values obtained for prediction metrics demonstrate the transfer learning potential, which is performed with extrapolation in traditional statistic and usually suffers from poor accuracy.

This work contributes to the field of cement nanocomposites design and quality control associated with identifying the effect of low dosages of engineered nanomaterials inclusion in reinforcement assessment; microstructure, and mechanical properties of cement-based composites can also be correlated to the fabrication, workability, and hydration in optimization tasks. The extensive use of statistics in the microstructure identification in the past decades was reasonable since computational strength was limited. However, technology evolution increases the necessity of materials scientists to adapt and improve their tools and data capacity for closing the gap of new ideas for design and applicability evaluation with less effort and need for resources. In this direction, Artificial Intelligence can provide a module for enabling fast, in-line, and real-time metrological characterization of nanoindentation data. Emphasis is located on classification of newly characterized data (specimen testing) based on a labelled database, which is promising to minimize the requirement for human effort in quality control and life assessment of Portland Cement formulations. The proposed microstructure analysis of Portland Cement using AI on nanoindentation data processing provided considerable proceedings; namely, classification reached an ultimate plateau value of 97.6% model accuracy using ANOVA SVC kernel and minimum F1 score of 95% in the five-class classification problem. Additionally, all approaches required a few seconds of computational time for clustering, training, and fitting. The high levels of accuracy hold promise for transfer learning potential and scalability of this methodology to expand prior obtained knowledge on new data. 

## Figures and Tables

**Table 1:** Material parameters determined by nanoindentation of hydrated cement phases.

Cement Phase	*E_r_* (GPa)	*H* (GPa)	Reference
Low stiffness phase	0–13	<0.4	[22,29,30,31,32]
Low density C–S–H	7–34	0.4–0.8	[2,3,15,18,19,22,23,29,30,31,32,33,34,35,36,37,38,39,40,41]
High density C–S–H	25-39	0.8–1.25	[3,15,18,19,22,23,29,30,31,32,36,37,38,40,42]
Portlandite (CH)	<35	1.31–1.66	[2,29,30,31,32,33,35,36,37,40,42]
Anisotropic Portlandite (CH)	≥99	≥2.8	[21,23,31,41,43]
Clinker	93–160	3–10	[3,17,21,22,30,31,37,40,41]

**Table 2:** Descriptive statistics of Portland Cement phases by implementing Probability Density Function (PDF) and k-means analysis.

	PDF Probability Peak	PDF skew	PDF *E_r_* (GPa)	Deviation (± GPa)	PDF Volume Fraction	k-Means *H* (GPa)	k-Means Deviation (± GPa)	k-Means *E_r_* (GPa)	k-Means Deviation (± GPa)	Counts	k-Means Volume Fraction
**LD C-S-H**	0.0190	−0.10	4	15	0.224	0.20	0.16	7.22	4.75	272	0.344
**HD C-S-H**	0.0240	−0.04	20	16	0.426	0.71	0.28	23.04	5.01	281	0.356
**CH**	0.0150	0.47	33	18	0.320	1.40	0.39	26.99	8.02	183	0.232
**Anisotropic CH**	0.0010	−0.33	61	13	0.014	2.67	0.61	54.16	11.53	43	0.054
**Clinker Interface**	0.0007	−0.44	90	20	0.016	5.86	1.66	88.35	10.11	11	0.014

**Table 3:** Statistic Metrics about KNN model.

KNN Metrics	CH	Anisotropic CH	Clinker Interface	HD C–S–H	LD C–S–H
Support (Counts–Test dataset)	26	27	7	44	62
Precision	0.500000	0.583333	0.333333	0.727273	1.000000
Recall	0.730769	0.259259	0.125000	0.909091	0.951613
F1	0.593750	0.358974	0.181818	0.808081	0.975207
Accuracy	0.754491				
MacroAvgPrecision	0.628788				
MacroAvgRecall	0.595147				
MacroAvgF1	0.583566				
MicroAvgPrecision	0.754491				
MicroAvgRecall	0.754491				
MicroAvgF1	0.754491				

**Table 4:** Statistic Metrics about Random Forest model.

RF Metrics	CH	Anisotropic CH	Clinker Interface	HD C–S–H	LD C–S–H
Support (Counts–Test dataset)	42	10	3	53	59
Precision	1.000000	0.833333	1.000000	0.963636	1.000000
Recall	0.904762	1.000000	1.000000	1.000000	1.000000
F1	0.950000	0.909091	1.000000	0.981482	1.000000
Accuracy	0.976048				
MacroAvgPrecision	0.959394				
MacroAvgRecall	0.980952				
MacroAvgF1	0.968115				
MicroAvgPrecision	0.976048				
MicroAvgRecall	0.976048				
MicroAvgF1	0.976048				

**Table 5:** Statistic Metrics about Radial Support Vector Classification (SVC) model.

SVC Radial Kernel Classification Metrics	CH	Anisotropic CH	Clinker Interface	HD C–S–H	LD C–S–H
Support (Counts–Test dataset)	39	11	3	56	58
Precision	0.973684	0.833333	1.000000	1.000000	0.983051
Recall	0.948718	0.909091	1.000000	0.982143	1.000000
F1	0.961039	0.869565	1.000000	0.990991	0.991453
Accuracy	0.976048				
MacroAvgPrecision	0.958014				
MacroAvgRecall	0.967990				
MacroAvgF1	0.962610				
MicroAvgPrecision	0.976048				
MicroAvgRecall	0.976048				
MicroAvgF1	0.976048				

**Table 6:** Statistic Metrics about Gaussian SVC model.

SVC Gaussian Kernel Classification Metrics	CH	Anisotropic CH	Clinker Interface	HD C–S–H	LD C–S–H
**Support (Counts–Test dataset)**	41	10	3	56	57
**Precision**	1.000000	0.833333	1.000000	0.981818	0.966102
**Recall**	0.926829	1.000000	1.000000	0.964286	1.000000
**F1**	0.962025	0.909091	1.000000	0.972973	0.982759
**Accuracy**	0.970060				
**MacroAvgPrecision**	0.956251				
**MacroAvgRecall**	0.978223				
**MacroAvgF1**	0.965370				
**MicroAvgPrecision**	0.970060				
**MicroAvgRecall**	0.970060				
**MicroAvgF1**	0.970060				

**Table 7:** Statistic Metrics about ANOVA SVC model.

SVC ANOVA Kernel Classification Metrics	CH	Anisotropic CH	Clinker Interface	HD C–S–H	LD C–S–H
Support (Counts–Test dataset)	40	11	3	56	57
Precision	1.000000	0.916667	1.000000	0.981818	0.966102
Recall	0.950000	1.000000	1.000000	0.964286	1.000000
F1	0.974359	0.956522	1.000000	0.972973	0.982759
Accuracy	0.976048				
MacroAvgPrecision	0.972917				
MacroAvgRecall	0.982857				
MacroAvgF1	0.977323				
MicroAvgPrecision	0.976048				
MicroAvgRecall	0.976048				
MicroAvgF1	0.976048				

## Appendix A

**Table A1:** R session info and Packages (accessed date: 2019-09-30).

Package	Version	Sour	ce		Package	Version	Sour	ce	
**abind**	1.4-5	CRAN	(R	3.6.0)	pillar	1.4.1	CRAN	(R	3.6.0)
**assertthat**	0.2.1	CRAN	(R	3.6.0)	pkgbuild	1.0.3	CRAN	(R	3.6.0)
**backports**	1.1.4	CRAN	(R	3.6.0)	pkgconfig	2.0.2	CRAN	(R	3.6.0)
**broom**	0.5.2	CRAN	(R	3.6.0)	pkgload	1.0.2	CRAN	(R	3.6.0)
**callr**	3.2.0	CRAN	(R	3.6.0)	plyr	1.8.4	CRAN	(R	3.6.0)
**caret**	6.0-84	CRAN	(R	3.6.1)	prettyunits	1.0.2	CRAN	(R	3.6.0)
**cellranger**	1.1.0	CRAN	(R	3.6.0)	pROC	1.15.3	CRAN	(R	3.6.1)
**class**	7.3-15	CRAN	(R	3.6.1)	processx	3.3.1	CRAN	(R	3.6.0)
**data.table**	1.12.2	CRAN	(R	3.6.0)	prodlim	2018.04.18	CRAN	(R	3.6.1)
**desc**	1.2.0	CRAN	(R	3.6.0)	ps	1.3.0	CRAN	(R	3.6.0)
**devtools**	2.2.0	CRAN	(R	3.6.1)	purrr	0.3.2	CRAN	(R	3.6.0)
**digest**	0.6.20	CRAN	(R	3.6.1)	R6	2.4.0	CRAN	(R	3.6.0)
**dplyr**	0.8.3	CRAN	(R	3.6.1)	randomForest	4.6-14	CRAN	(R	3.6.1)
**DT**	0.9	CRAN	(R	3.6.0)	Rcpp	1.0.1	CRAN	(R	3.6.0)
**e1071**	1.7-2	CRAN	(R	3.6.1)	readr	1.3.1	CRAN	(R	3.6.0)
**ellipsis**	0.2.0.1	CRAN	(R	3.6.1)	readxl	1.3.1	CRAN	(R	3.6.1)
**ggplot2**	3.1.1	CRAN	(R	3.6.0)	reshape2	1.4.3	CRAN	(R	3.6.0)
**glue**	1.3.1	CRAN	(R	3.6.0)	rJava	0.9-11	CRAN	(R	3.6.0)
**htmltools**	0.3.6	CRAN	(R	3.6.0)	rlang	0.4.0	CRAN	(R	3.6.1)
**htmlwidgets**	1.3	CRAN	(R	3.6.0)	scales	1.0.0	CRAN	(R	3.6.0)
**httr**	1.4.0	CRAN	(R	3.6.0)	segmented	1.0-0	CRAN	(R	3.6.0)
**kernlab**	0.9-27	CRAN	(R	3.6.0)	sessioninfo	1.1.1	CRAN	(R	3.6.0)
**labeling**	0.3	CRAN	(R	3.6.0)	shiny	1.3.2.9001	Gith	ub	(rstudio/shiny@89bd7e9)
**lattice**	0.20-38	CRAN	(R	3.6.0)	sjlabelled	1.1.1	CRAN	(R	3.6.1)
**lava**	1.6.6	CRAN	(R	3.6.1)	stringi	1.4.3	CRAN	(R	3.6.0)
**lazyeval**	0.2.2	CRAN	(R	3.6.0)	stringr	1.4.0	CRAN	(R	3.6.0)
**lubridate**	1.7.4	CRAN	(R	3.6.0)	survival	2.44-1.1	CRAN	(R	3.6.0)
**magic**	1.5-9	CRAN	(R	3.6.0)	testthat	2.2.1	CRAN	(R	3.6.1)
**magrittr**	1.5	CRAN	(R	3.6.0)	tibble	2.1.3	CRAN	(R	3.6.0)
**MASS**	7.3-51.4	CRAN	(R	3.6.0)	tidyr	0.8.3	CRAN	(R	3.6.0)
**Matrix**	1.2-17	CRAN	(R	3.6.0)	tidyselect	0.2.5	CRAN	(R	3.6.0)
**mclust**	5.4.5	CRAN	(R	3.6.1)	tidyverse	1.2.1	CRAN	(R	3.6.0)
**mixtools**	1.1.0	CRAN	(R	3.6.1)	timeDate	3043.102	CRAN	(R	3.6.0)
**mlbench**	2.1-1	CRAN	(R	3.6.1)	usethis	1.5.0	CRAN	(R	3.6.0)
**MLmetrics**	1.1.1	CRAN	(R	3.6.1)	withr	2.1.2	CRAN	(R	3.6.0)
**ModelMetrics**	1.2.2	CRAN	(R	3.6.1)	xlsx	0.6.1	CRAN	(R	3.6.0)
**modelr**	0.1.4	CRAN	(R	3.6.0)	xlsxjars	0.6.1	CRAN	(R	3.6.0)
**pdfCluster**	1.0-3	CRAN	(R	3.6.1)	xml2	1.2.0	CRAN	(R	3.6.0)

**Table A2:** Detailed performance results of KNN hyperparameter tuning.

k	Error	Dispersion	k	Error	dispersion	k	Error	Dispersion
**1**	0.667742	0.047836	**15**	0.569765	0.042177	**28**	0.592217	0.058813
**2**	0.658013	0.048698	**16**	0.589094	0.050706	**29**	0.598566	0.062328
**3**	0.603405	0.083304	**17**	0.57788	0.04982	**30**	0.590502	0.071531
**4**	0.601843	0.073970	**18**	0.584485	0.053367	**31**	0.579186	0.080118
**5**	0.587276	0.075722	**19**	0.582693	0.058426	**32**	0.598464	0.068451
**6**	0.584101	0.054559	**20**	0.586022	0.055453	**33**	0.580901	0.065523
**7**	0.569739	0.063925	**21**	0.605146	0.048521	**34**	0.57934	0.067627
**8**	0.592217	0.057005	**22**	0.579519	0.048427	**35**	0.574552	0.065912
**9**	0.581055	0.071392	**23**	0.608321	0.053482	**36**	0.593907	0.054005
**10**	0.577829	0.059211	**24**	0.582617	0.040259	**37**	0.58743	0.066458
**11**	0.576114	0.043615	**25**	0.608244	0.047015	**38**	0.587353	0.068217
**12**	0.558577	0.045023	**26**	0.606682	0.044701	**39**	0.59703	0.060446
**13**	0.577803	0.026752	**27**	0.595366	0.060007	**40**	0.585689	0.068017
**14**	0.58254	0.056609						

**Table A3:** Confusion matrix of the testing dataset and the predicted Portland Cement Phases by KNN model.

KNN Classification	CH	Anisotropic CH	Clinker Interface	HD C-S-H	LD C-S-H
CH	19	1	0	6	0
Anisotropic CH	13	7	2	5	0
Clinker interface	2	4	1	1	0
HD C-S-H	4	0	0	40	0
LD C-S-H	0	0	0	3	59

**Table A4:** Confusion matrix of the testing dataset and the predicted Portland Cement Phases by random forrest model.

RF Classification	CH	Anisotropic CH	Clinker Interface	HD C-S-H	LD C-S-H
CH	38	2	0	2	0
Anisotropic CH	0	10	0	0	0
Clinker interface	0	0	3	0	0
HD C-S-H	0	0	0	53	0
LD C-S-H	0	0	0	0	59

**Table A5:** Detailed performance results of Radial Support Vector Classification (SVC) kernel hyperparameter tuning.

#	Gamma	Cost	Error	Dispersion	#	Gamma	Cost	Error	Dispersion
**1**	0.01	1	0.121941	0.048335	**21**	0.01	400	0.036918	0.02526
**2**	0.1	1	0.072248	0.035021	**22**	0.1	400	0.052970	0.036494
**3**	0.5	1	0.083436	0.032799	**23**	0.5	400	0.083410	0.036922
**4**	2	1	0.125166	0.049255	**24**	2	400	0.104327	0.047335
**5**	4	1	0.146032	0.042723	**25**	4	400	0.131618	0.034375
**6**	0.01	10	0.051357	0.014704	**26**	0.01	1000	0.040143	0.026622
**7**	0.1	10	0.052919	0.033045	**27**	0.1	1000	0.056144	0.038113
**8**	0.5	10	0.086610	0.034644	**28**	0.5	1000	0.081797	0.034984
**9**	2	10	0.110701	0.045551	**29**	2	1000	0.104327	0.047335
**10**	4	10	0.134793	0.036901	**30**	4	1000	0.131618	0.034375
**11**	0.01	50	0.040118	0.028672	**31**	0.01	4000	0.030492	0.028863
**12**	0.1	50	0.048157	0.026341	**32**	0.1	4000	0.049770	0.039092
**13**	0.5	50	0.083436	0.035396	**33**	0.5	4000	0.086610	0.033016
**14**	2	50	0.107501	0.045313	**34**	2	4000	0.104327	0.047335
**15**	4	50	0.134793	0.036901	**35**	4	4000	0.131618	0.034375
**16**	0.01	100	0.038530	0.024244	**36**	0.01	8000	0.033769	0.030845
**17**	0.1	100	0.051382	0.032078	**37**	0.1	8000	0.048182	0.032232
**18**	0.5	100	0.089836	0.033124	**38**	0.5	8000	0.086610	0.033016
**19**	2	100	0.102739	0.048516	**39**	2	8000	0.104327	0.047335
**20**	4	100	0.136380	0.034517	**40**	4	8000	0.131618	0.034375

**Table A6:** Confusion matrix of the testing dataset and the predicted Portland Cement Phases by Radial SVC model.

SVC Radial Kernel Classification	CH	Anisotropic CH	Clinker Interface	HD C-S-H	LD C-S-H
CH	37	2	0	0	0
Anisotropic CH	1	10	0	0	0
Clinker interface	0	0	3	0	0
HD C-S-H	0	0	0	55	1
LD C-S-H	0	0	0	0	58

**Table A7:** Confusion matrix of the testing dataset and the predicted Portland Cement Phases by Gaussian SVC model.

SVC Gaussian Kernel Classification	CH	Anisotropic CH	Clinker Interface	HD C-S-H	LD C-S-H
CH	38	2	0	1	0
Anisotropic CH	0	10	0	0	0
Clinker interface	0	0	3	0	0
HD C-S-H	0	0	0	54	2
LD C-S-H	0	0	0	0	57

**Table A8:** Confusion matrix of the testing dataset and the predicted Portland Cement Phases by ANOVA SVC model.

SVC ANOVA Kernel Classification	CH	Anisotropic CH	Clinker Interface	HD C-S-H	LD C-S-H
CH	38	1	0	1	0
Anisotropic CH	0	11	0	0	0
Clinker interface	0	0	3	0	0
HD C-S-H	0	0	0	54	2
LD C-S-H	0	0	0	0	57
